# LncRNA RP11-86H7.1 promotes airway inflammation induced by TRAPM2.5 by acting as a ceRNA of miRNA-9-5p to regulate NFKB1 in HBECS

**DOI:** 10.1038/s41598-020-68327-1

**Published:** 2020-07-14

**Authors:** Jun Zhao, Jinding Pu, Binwei Hao, Lingmei Huang, Jinglong Chen, Wei Hong, Yumin Zhou, Bing Li, Pixin Ran

**Affiliations:** 1 State Key Laboratory of Respiratory Diseases, National Clinical Research Center for Respiratory Diseases, Guangzhou Institute of Respiratory Health, The First Affiliated Hospital of Guangzhou Medical University, 195 Dongfeng Xi Road, Guangzhou, 510182 Guangdong China; 2 Department of Geriatrics, National Clinical Key Specialty, Guangzhou First People’s Hospital, School of Medicine, South China University of Technology, Guangzhou, China; 3 Guangzhou First People’s Hospital, Guangzhou Medical University, Guangzhou, Guangdong China; 4 GMU-GIBH Joint School of Life Sciences, Guangzhou Medical University, Guangzhou, Guangdong China; 5 The First People’s Hospital of YueYang, YueYang, Hunan China

**Keywords:** Molecular biology, Environmental sciences, Biomarkers, Diseases, Molecular medicine, Pathogenesis

## Abstract

Traffic-related air pollution particulate matter 2.5 (TRAPM2.5), is involved in chronic obstructive pulmonary disease (COPD), which is characterized by airway inflammation. Specifically, these harmful particles or gases can increase chronic airway inflammation. Some recent studies have shown that lncRNAs are closely related to COPD and participate in the regulation of airway inflammation. However, the precise mechanisms remain unknown. In the present study, we investigated the effect of TRAPM2.5 on airway inflammation in human bronchial epithelial cells (HBECs) and the underlying mechanisms mediated by a lncRNA. After exposure to TRAPM2.5, the novel lncRNA RP11-86H7.1 was markedly upregulated in HBECs. Functional assays indicated that the lncRNA RP11-86H7.1 was required for the TRAPM2.5-induced expression of inflammatory factors in HBECs. A mechanistic study demonstrated that lncRNA RP11-86H7.1 might participate in TRAPM2.5-induced inflammatory responses by activating the NF-κB signaling pathway. Moreover, the lncRNA RP11-86H7.1 can promote the inflammatory response by acting as a competing endogenous RNA of miR-9-5p, reversing the inhibitory effect of its target gene NFKB1, and sustaining NF-κB activation. In summary, our study elucidates the pro-inflammatory roles of the lncRNA RP11-86H7.1–miR-9-5p–NFKB1 regulatory network in airway inflammation induced by TRAPM2.5 and indicates that the components of this network might serve as novel diagnostic biomarkers and potential therapeutic targets.

## Introduction

It is well known that smoking is the most important risk factor for chronic obstructive lung disease (COPD), but a growing number of studies have shown that other environmental exposures, such as biofuel exposure^1–3^ and air pollution^4,5^, also play an important role in the development of COPD. These harmful particles or gases can increase chronic airway inflammation and the oxidation-antioxidant imbalance, leading to abnormal airway and/or alveoli, aggravated respiratory symptoms and airflow limitation in patients with COPD^6^. Our recent findings revealed that exposure to higher particulate matter (PM) concentrations, particularly PM2.5, is associated with an increased prevalence of COPD and declined respiratory function^7^.

Due to China’s rapid urbanization and increasing industrialization, PM2.5 from traffic-related air pollution (TRAP) generated by motor vehicle exhaust has become a major source of air pollutant emissions^8^. Many epidemiological studies performed in recent years have revealed that TRAPM2.5 can increase the incidence of COPD, the risk of acute exacerbation and the rate of mortality and decrease lung function^9–14^. However, whether TRAPM2.5 directly leads to the pathogenesis of COPD remains unclear. The published findings were obtained from epidemiological studies, and experiments and studies on the specific pathogenesis have not been performed.

Unlike messenger RNAs, noncoding RNAs (ncRNAs) in the genome are called “dark matter” or “junk RNA” because they cannot encode proteins. According to their nucleotide lengths, ncRNAs can be classified into three groups: housekeeping RNAs, short ncRNAs (< 200 nucleotides), such as microRNAs (miRNAs, 18–24 nucleotides), and long ncRNAs (lncRNAs, > 200 nucleotides), which have important functions in various aspects of cell biology and life processes^15^. Increasing evidence has uncovered the indispensable function of miRNAs in respiratory diseases, such as COPD, lung cancer, pneumonia, asthma, and pulmonary fibrosis^16,17^. In contrast to miRNAs, much less is known regarding the roles of the many thousands of lncRNAs in the field of COPD. However, lncRNAs are involved in the regulation of gene expression at multiple levels in the form of RNA, and the abnormal expression of lncRNAs is closely related to respiratory diseases. These findings are likely to open an exciting and fertile new area of scientific discovery in the field of COPD.

The airway inflammatory response induced by harmful particles or gases is the main cause of the pathogenesis and development of COPD. After entering the airway, the harmful particles first contact the bronchial epithelial cells, induce changes in the bronchial epithelial phenotype and then stimulate these cells to secrete a large number of cytokines and inflammatory mediators. This secretion causes an inflammatory cascade and thereby aggravates airway inflammation and lung damage. To elucidate the key role of lncRNAs in the pathological process of airway inflammation caused by TRAPM2.5 and to analyze their relationship with the occurrence and development of COPD, we collected and prepared TRAPM2.5 and stimulated human primary bronchial epithelial cells with TRAPM2.5, and the differential expression profiles of lncRNAs were then analyzed by high-throughput gene microarray technology. Through gain and loss of function approaches, we found that the lncRNA RP11-86H7.1 has important functions in the inflammatory response of HBECs to TRAPM2.5 and further revealed that the lncRNA RP11-86H7.1 can serve as a sponge for miR-9-5p through the competing endogenous RNA (ceRNA) mechanism to inhibit the silencing effect of miR-9-5p on NFKB1 and upregulate the expression of NFKB1 and inflammatory factors and thus promote TRAPM2.5-induced airway inflammation.

## Results

### TRAPM2.5 induces the expression of inflammatory factors in HBECs through activation of the NF-κB signaling pathway

**Figure 1:**
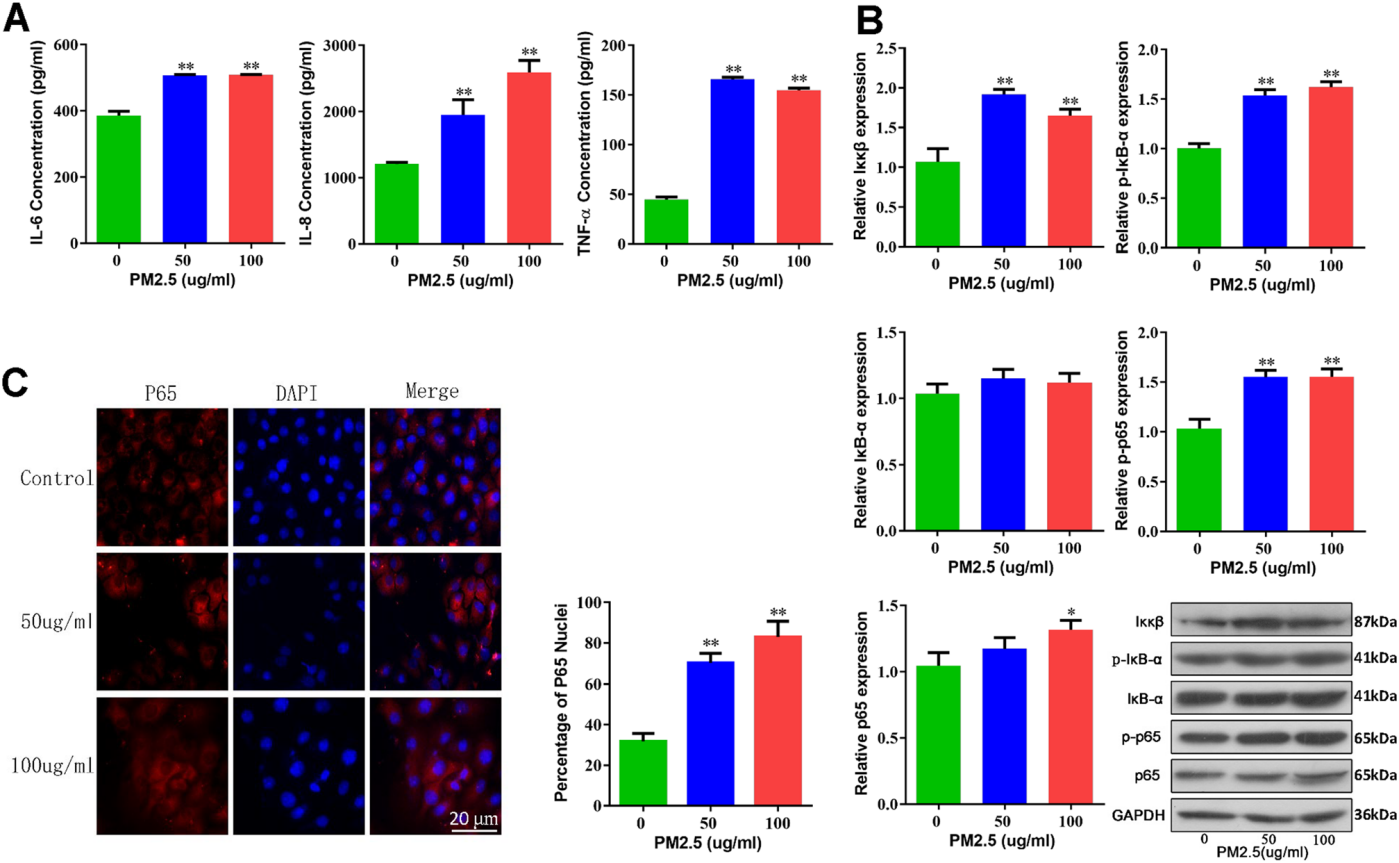
Effects of TRAPM2.5 on the inflammatory response of human bronchial epithelial cells. (**A**) ELISAs analysis of IL-6, IL-8, and TNF-α levels in HBECs treated with 0, 50, and 100 μg/ml TRAPM2.5 for 48 h. (**B**) Western blotting analysis of Iκκβ, p-IκB-α, IκB-α, p-p65 and p65 in HBECs treated with 0, 50, and 100 μg/ml TRAPM2.5 for 48 h. GAPDH was used as an internal control. (**C**) Immunofluorescence microscopy showing the localization and expression of p65 in HBECs treated with 0, 50, and 100 μg/ml TRAPM2.5 for 48 h. Original magnification, × 200. Scale bar 20 μm. The data are shown as the means ± standard deviations (n = 3). The statistical significance of the data was assessed by Student’s *t* test. *P < 0.05, **P < 0.01.

First, we collected and prepared TRAPM2.5 as previously described^18^. It is well known that the inflammatory response of airway epithelial cells to harmful particles or gases is closely related to the upregulation of many inflammatory factors, including IL-6, IL-8, and TNF-α^19–21^. To evaluate the inflammatory response of HBECs exposed to 100 μg/ml TRAPM2.5 for 48 h, the levels of the inflammatory factors IL-6, IL-8, and TNF-α were detected by ELISA (Fig. 1A). The results showed that TRAPM2.5 promoted the levels of inflammatory factors and thus induced an inflammatory response in HBECs.

NF-κB plays a key regulatory role in the PM-induced inflammatory response and is closely related to IL-6, IL-8 and TNF-α^22–24^. We subsequently detected the protein levels of p65, p-p65, IκB-α, p-IκB-α, and Iκκβ by Western blotting and found that the p65, p-p65, p-IκB-α, and Iκκβ protein levels were significantly increased after treatment with 100 μg/ml TRAPM2.5 for 48 h (Fig. 1B).

The nuclear translocation of p65 is a necessary condition for activation of the NF-κB signaling pathway^25^. To further verify the activation of the NF-κB signaling pathway, the nuclear translocation of p65 was detected by immunofluorescence. After TRAPM2.5 stimulation for 48 h, the cells were observed under a fluorescence microscope: the target p65 was stained with red fluorescence, and the DAPI-stained nuclei exhibited a blue fluorescence (Fig. 1C). The results indicated that TRAPM2.5 stimulation increased the translocation of p65 into the nucleus, which demonstrated that the NF-κB signaling pathway was indeed activated.

### Differential expression profiles of lncRNAs and mRNAs in TRAPM2.5-treated HBECs and bioinformatics analysis

To identify the TRAPM25-induced changes in lncRNA expression in HBECs, the total RNA from cells before and 48 h after TRAPM2.5 treatment was obtained and subjected to 4** × **44 K Agilent Whole Human Genome Oligo Microarray (Agilent, USA) analysis. This process was repeated three times for each sample. According to the data obtained, the following screening criterion was set: genes with fold-change > 2 were considered differentially expressed genes. The gene microarray analysis showed that 1698 lncRNAs were significantly upregulated and 2,321 lncRNAs were significantly downregulated in HBECs after TRAPM2.5 treatment for 48 h compared with the control group (DMSO group) (Supplementary Fig. S1A). Among the different mRNAs, 1,405 mRNAs were significantly upregulated, and 597 mRNAs were significantly downregulated. The minimum and maximum difference multipliers were 2.0 and 241.3, respectively. The lncRNA-regulated mRNAs were analyzed by bioinformatics, and GO and KEGG enrichment analyses were conducted to analyze the biological processes, molecular functions, cell compositions and cell signaling pathways of these mRNAs (Supplementary Fig. S1B–D).

### Screening and validation of lncRNAs relate to the NF-κB signaling pathway

**Figure 2:**
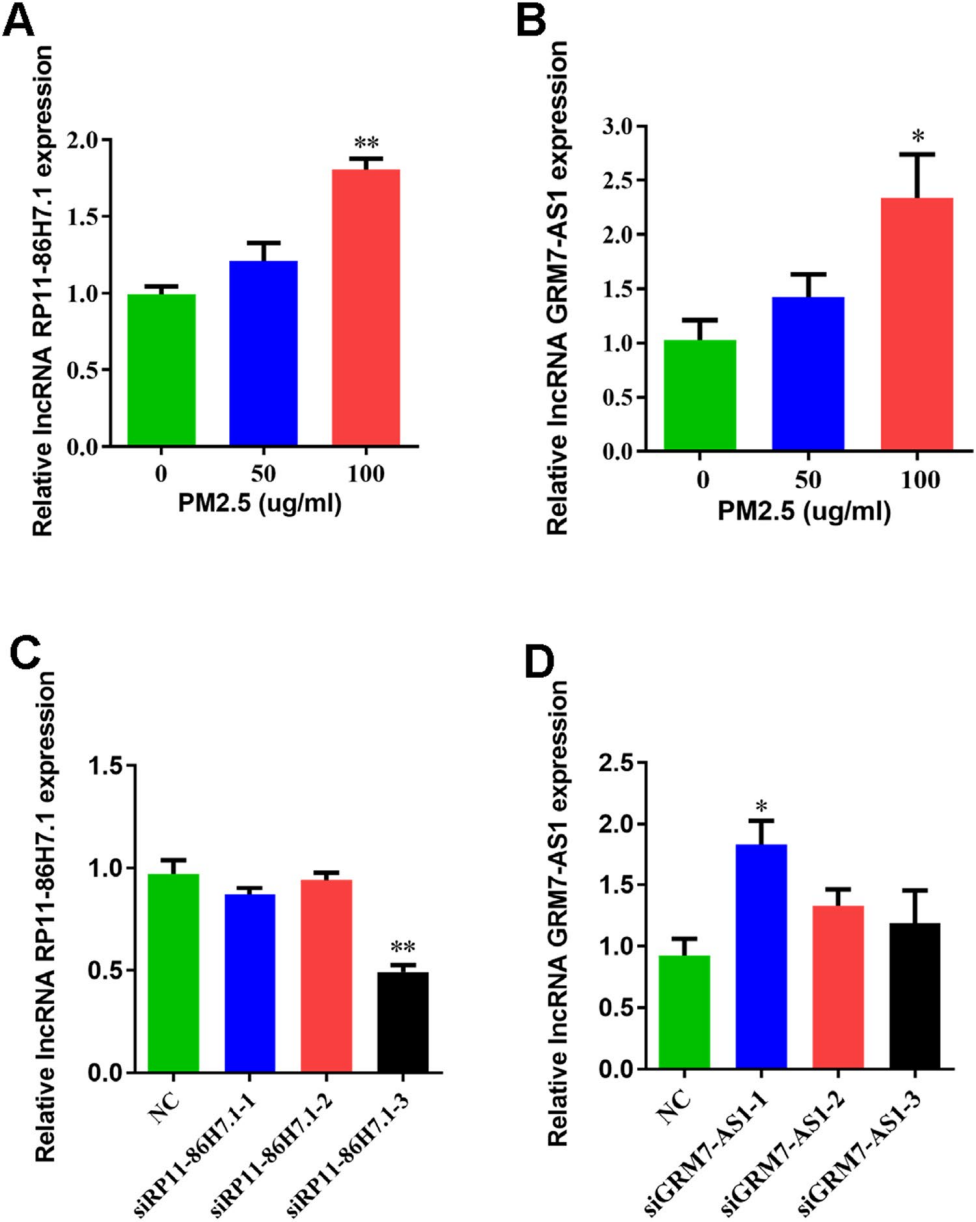
Screening and validation of lncRNAs related to the NF-κB signaling pathway. (**A**) Q-PCR analysis of lncRNA RP11-86H7.1 expression level in HBECs after TRAPM2.5 treatment. (**B**) Q-PCR analysis of lncRNA GRM7-AS1 expression level in TRAPM2.5-treated HBECs. (**C**) Q-PCR analysis of the three target sequences by RNA interference of the lncRNA RP11-86H7.1. (**D**) Q-PCR analysis of the three target sequences by RNA interference of the lncRNA GRM7-AS1. The data are shown as the means ± standard deviations (n = 3). The statistical significance of the data was assessed by Student’s *t* test. *P < 0.05, **P < 0.01. siRNA RP11-86H7.1-1: interference fragment 1 of lncRNA RP11-86H7.1; siRNA RP11-86H7.1-2: interference fragment 2 of lncRNA RP11-86H7.1; siRNA RP11-86H7.1-3: interference fragment 3 of lncRNA RP11-86H7.1; siRNA GRM7-AS1-1: interference fragment 1 of lncRNA GRM7-AS1; siRNA GRM7-AS1-2: interference fragment 2 of lncRNA GRM7-AS1; siRNA GRM7-AS1-3: interference fragment 3 of GRM7-AS1.

First, using various bioinformatics methods, including analyses of the KEGG, miRTarBase and StarBase databases, GO functional analysis, and miRNAda software, we predicted three lncRNAs related to the NF-κB inflammatory signaling pathway: RP11-86H7.1, GRM7-AS1, and RP11-39M21.1. To further verify the microarray results, a Q-PCR analysis was performed, and the results revealed that exposure to increasing concentrations of TRAPM2.5 (0, 50, and 100 µg/ml) for 48 h induced gradual increases in the lncRNA RP11-86H7.1 and GRM7-AS1 mRNA levels, but RP11-39M21.1 was not detectable. Compared with those found in the control group (0 µg/ml), the lncRNA RP11-86H7.1 and GRM7-AS1 mRNA levels significantly increased after stimulation with 100 µg/ml TRAPM2.5 (Fig. 2A,B). In addition, the CT value of the lncRNA RP11-39M21.1 was too high and beyond the detection range, which might be due to low gene expression in the sample, and thus, no CT data were obtained. Three siRNA interference fragments each were screened for their effects on RP11-86H7.1 and GRM7-AS1 by Q-PCR. The results suggested no significant interference effect by the three siRNAs targeting lncRNA GRM7-AS1 but did reveal a significant interference effect of interference fragment 3 on the lncRNA RP11-86H7.1 (Fig. 2C,D). Therefore, the lncRNA RP11-86H7.1 was selected as the target lncRNA in the subsequent studies.

### LncRNA RP11-86H7.1 is required in the TRAPM2.5-induced inflammatory response of HBECs

**Figure 3:**
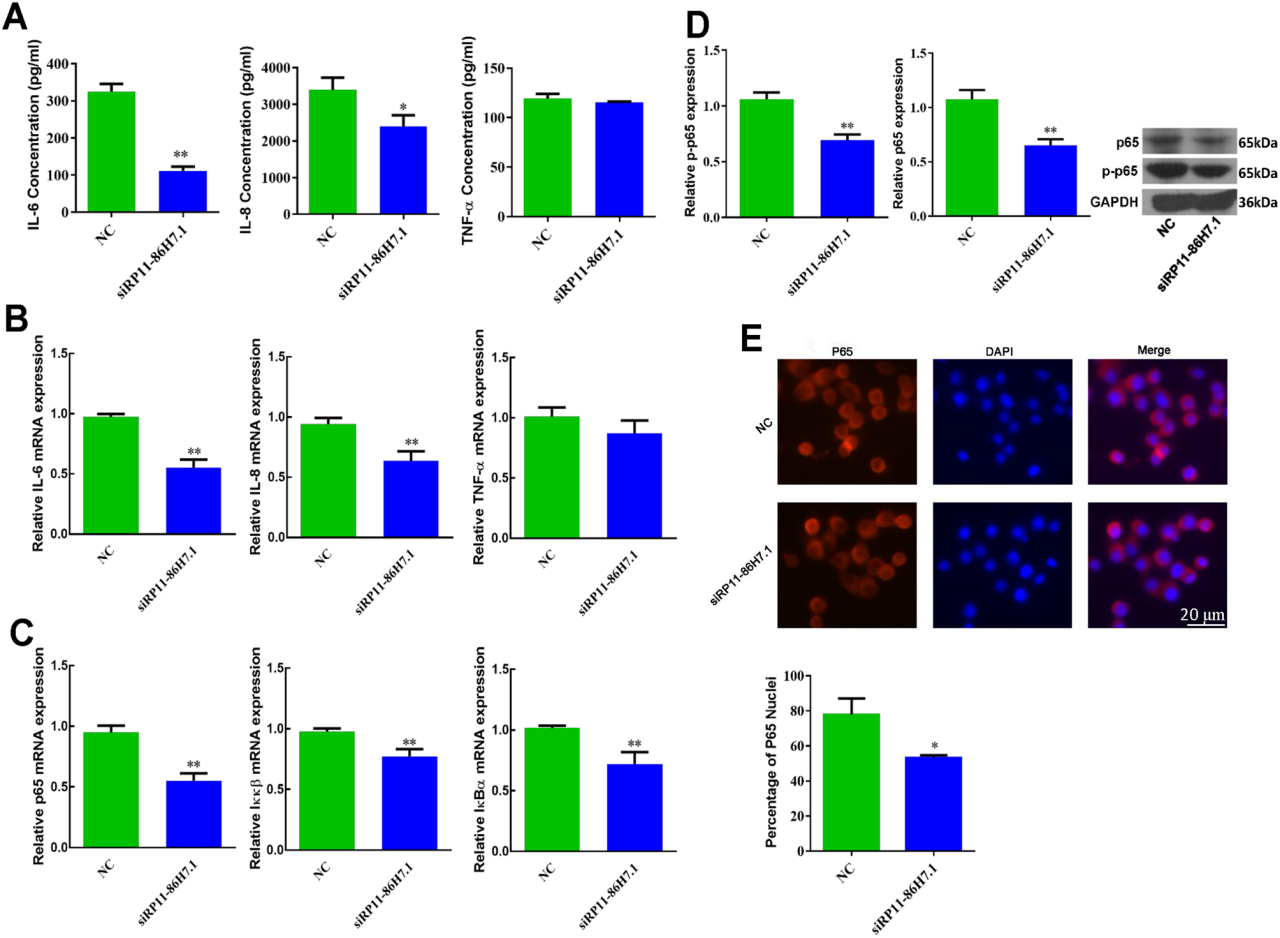
The lncRNA RP11-86H7.1 plays a necessary role in the TRAPM2.5-induced inflammatory response of HBECs. (**A**) ELISAs analysis of IL-6, IL-8, and TNF-α levels in HBECs treated with 100 μg/ml TRAPM2.5 after knockdown of the lncRNA RP11-86H7.1. (**B**) Q-PCR analysis of the IL-6, IL-8, and TNF-α mRNA expression levels in HBECs treated with 100 μg/ml TRAPM2.5 after knockdown of the lncRNA RP11-86H7.1. (**C**) Q-PCR analysis of the p65, Iκκβ, and IκBα mRNA expressions levels in HBECs treated with 100 μg/ml TRAPM2.5 after knockdown of the lncRNA RP11-86H7.1. (**D**) Western blotting analysis of the protein levels of p-p65 and p65 in HBECs treated with 100 μg/ml TRAPM2.5 after knockdown of the lncRNA RP11-86H7. GAPDH was used as an internal control. (**E**) Immunofluorescence microscopy showing the localization and expression of p65 in HBECs treated with 100 μg/ml TRAPM2.5 after knockdown of the lncRNA RP11-86H7. Original magnification, × 200. Scale bar 20 μm. The data are shown as the means ± standard deviations (n = 3). The statistical significance of the data was assessed by Student’s *t* test. *P < 0.05, **P < 0.01.

In HBECs, the expression of the lncRNA RP11-86H7.1 was significantly elevated after stimulation with 100 µg/ml TRAPM2.5, which suggested that the lncRNA RP11-86H7.1 might participate in the TRAPM2.5-induced inflammatory response in HBECs. The cells were transfected with siRNA specific for the lncRNA RP11-86H7.1 and then stimulated with 100 µg/ml TRAPM2.5 for 48 h. Knockdown of the lncRNA RP11-86H7.1 decreased the TRAPM2.5-induced expression of the inflammatory factors IL-6, IL-8, and the levels of p65, Iκκβ, IκBα mRNA in HBECs (Fig. 3A–C). Moreover, the p65 and p-p65 protein levels were significantly reduced after treatment with 100 μg/ml TRAPM2.5 for 48 h (Fig. 3D). Immunofluorescence also indicated that knockdown of the lncRNA RP11-86H7.1 inhibited the translocation of p65 into the nucleus, which demonstrated that the NF-κB signaling pathway was indeed suppressed (Fig. 3E).

### Full-length coding ability, subcellular distribution and coexpression network characteristics of the lncRNA RP11-86H7.1

**Figure 4:**
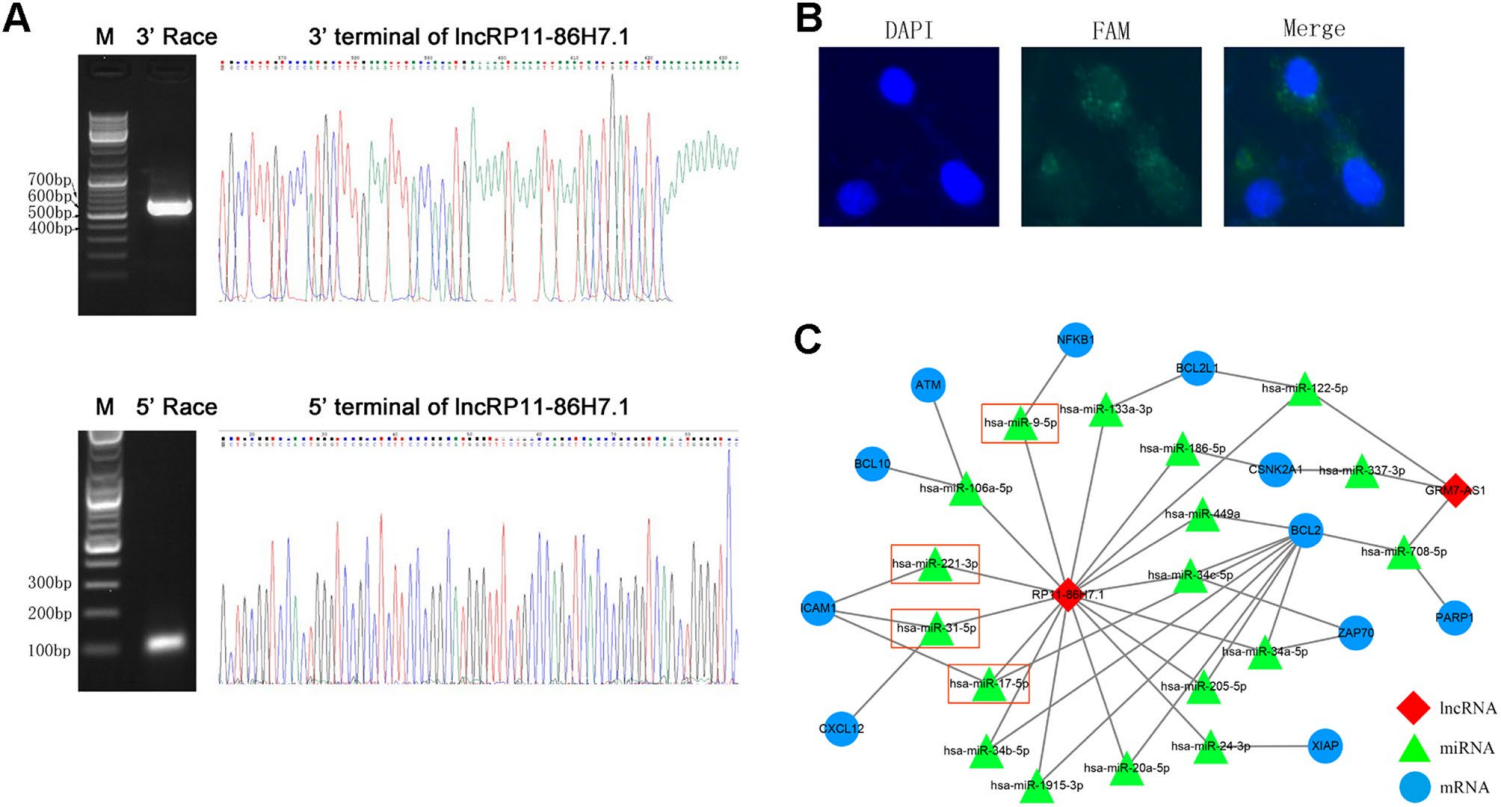
Full-length sequence, coding ability, subcellular distribution and coexpression network characteristics of the lncRNA RP11-86H7.1. (**A**) 5′-RACE and 3′-RACE assays were performed to determine the transcriptional initiation and termination sites of the lncRNA RP11-86H7.1. Left, representative images of the PCR products obtained by 5′-RACE and 3′-RACE. Right, the sequence of the second-round PCR products revealed the boundary between the universal anchor primer and the lncRNA RP11-86H7.1 sequence. (**B**) FISH analysis of the subcellular distribution of the lncRNA RP11-86H7.1 in 16HBE cells. Scale bar 20 μm. (**C**) Co-expression network characteristics of the lncRNA RP11-86H7.1. FAM refers to carboxyfluorescein, FAM-labeled lncRNA RP11-86H7.1 was stained green (FAM), and nuclei was stained blue with 6-diamidino-2-phenylindole (DAPI).

First, we obtained the length of the lncRNA RP11-86H7.1 by 5′ and 3′ RACE (Fig. 4A, Supplementary Table S1). Moreover, FISH revealed that the lncRNA RP11-86H7.1 was distributed in the cytoplasm (Fig. 4B). To further determine the role of the lncRNA RP11-86H7.1, we constructed a map of the lncRNA-miRNA-mRNA co-expression network using Cytoscape software and found that four miRNAs (miR-17-5p, miR-221-3p, miR-31-5p, and miR-9-5p) were associated with the NF-κB signaling pathway and might be sponged by the lncRNA RP11-86H7.1 (Fig. 4C).

### MiR-9-5p suppresses the inflammatory factors and the NF-κB signaling pathway in 16HBE cells

**Figure 5:**
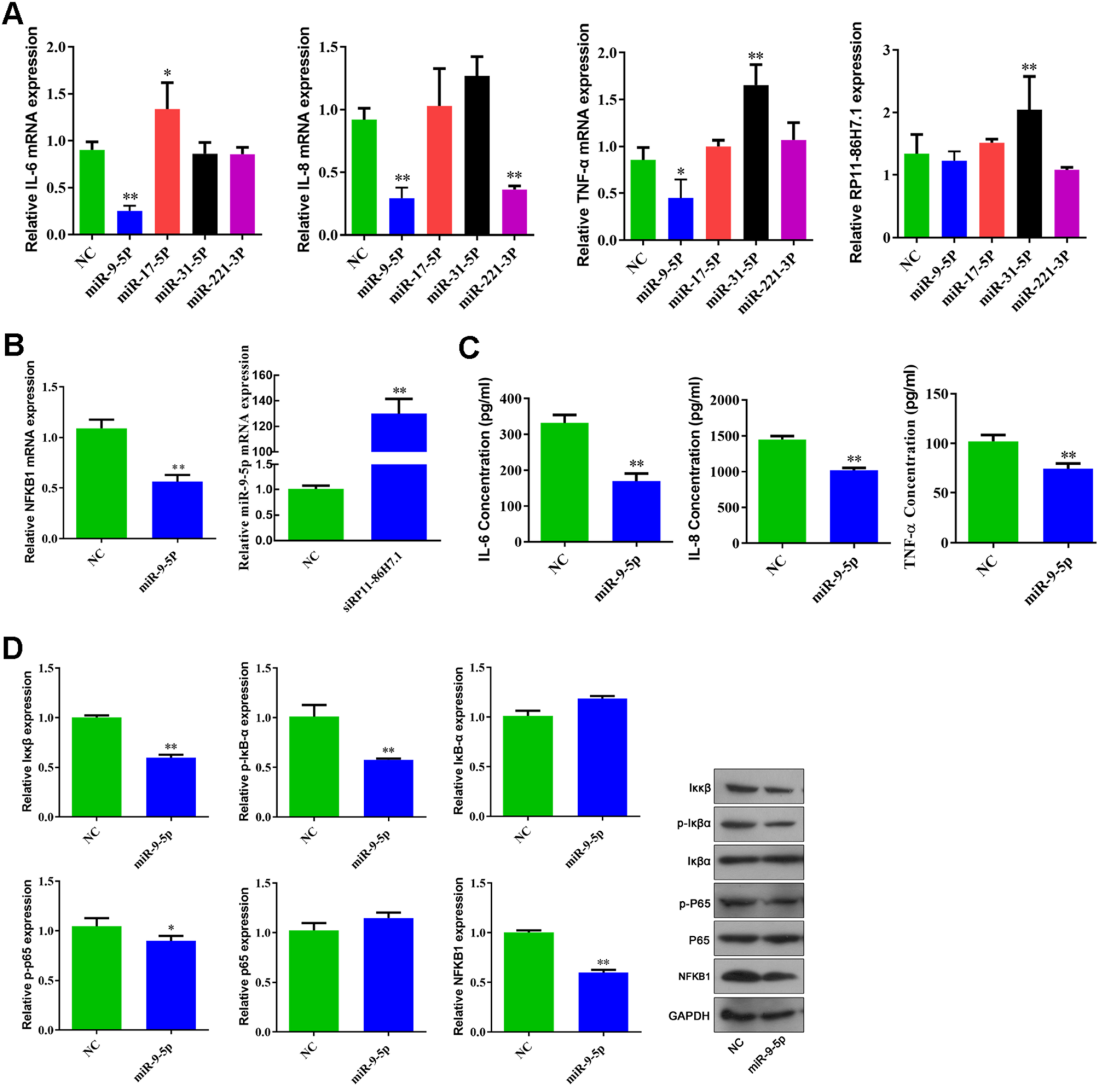
miR-9-5p suppresses inflammatory factors and the NF-κB signaling pathway in 16HBE cells. (**A**) Q-PCR analysis of the IL-6, IL-8, TNF-α and lncRNA RP11-86H7.1 mRNA levels after miRNA (miR-9-5p, miR-17-5p, miR-31-5p, and miR-221-3p) mimic transfection. (**B**) Q-PCR analysis of NFKB1 levels after miR-9-5p mimic transfection and miR-9-5p mRNA levels after knockdown of the lncRNA RP11-86H7.1. (**C**) ELISA analysis of IL-6, IL-8, and TNF-α levels in 16HBE cells after miR-9-5p mimic transfection. (**D**) Western blotting analysis of the protein levels of Iκκβ, p-IκB-α, IκB-α, p-p65, p65 and NFKB1 after miR-9-5p mimic transfection. GAPDH was used as an internal control. The data are shown as the means ± standard deviations (n = 3). The statistical significance of the data was assessed by Student’s *t* test. *P < 0.05, **P < 0.01.

To elucidate the roles of miRNAs (miR-17-5p, miR-221-3p, miR-31-5p, and miR-9-5p) in the inflammatory response of 16HBE cells, the cells were transiently transfected with miRNA mimics. The results showed that only miR-9-5p mimic suppressed the expression of IL-6, IL-8, TNF-α, p-IκB-α, p-p65, and Iκκβ (Fig. 5A–D). We also found that the overexpression of miR-9-5p inhibited the expression of NFKB1, whereas knockdown of the lncRNA RP11-86H7.1 significantly increased the expression of miR-9-5p (Fig. 5B).

### LncRNA RP11-86H7.1 as a ceRNA and competitively absorbs miR-9-5p with NFKB1

**Figure 6:**
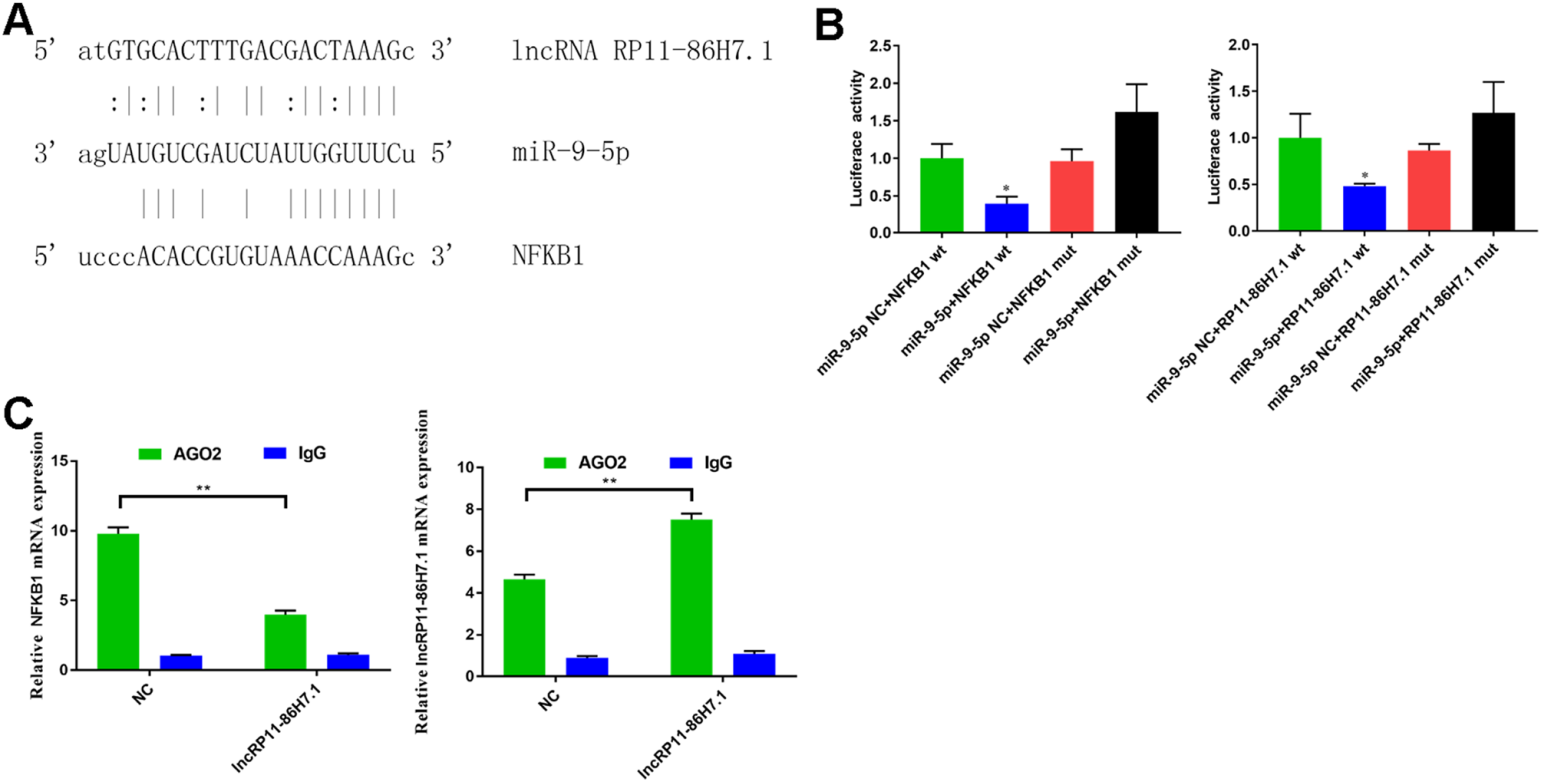
LncRNA RP11-86H7.1 acts as a ceRNA and competitively absorbs miR-9-5p with NFKB1. (**A**) LncRNA RP11-86H7.1, miR-9-5p and NFKB1 binding sites predicted by miRanda. (**B**) The luciferase activities in 293 T cells co-transfected with wild-type (wt) and mutant (mut) NFKB1 or RP11-86H7.1 plasmid together with miR-9-5p mimic or miR-9-5p negative control (NC). Luciferase activity was detected 24 h after transfection using the dual luciferase assay. Data are presented as the relative ratio of renilla luciferase activity and firefly luciferase activity. (**C**) The real-time PCR results of the RIP based on Ago2 showed that lncRNA RP11-86H7.1 can compete with the NFKB1 for the binding of miR-9-5p. IgG was used as negative control. The data are shown as the means ± standard deviations (n = 3). The statistical significance of the data was assessed by Student’s t test. *P < 0.05, **P < 0.01.

It is well known that lncRNAs can act as competing endogenous RNAs (ceRNAs) to protect mRNAs from degradation by competing for their targeting microRNAs. We thus investigated whether the lncRNA RP11-86H7.1 also plays such a role. First, using bioinformatics (miRanda) tools, we found that miR-9-5p can directly bind to both the lncRNA RP11-86H7.1 and NFKB1 (Fig. 6A). Second, the dual-luciferase reporter assay was applied to verify the direct interaction between miR-9-5p and 3′-UTR of NFKB1 and LncRNA RP11-86H7.1. When wild-type NFKB1 3′ UTR reporter vector and miR-9-5p mimic were co-transfected into 293 T cells, cell luciferase activity was significantly decreased, while the NFKB1 mutant in the miR-9-5p binding sites reversed this effect. It was show that miR-9-5p can target NFKB1 (Fig. 6B). Similarly, we also observed co-transfection with pmirGLO-RP11-86H7.1-wt vector and miR-9-5p mimic obviously inhibited cell luciferase activity, while the RP11-86H7.1 mutant revoked this effect (Fig. 6B). These results proved that miR-9-5p can bind to both lncRNA RP11-86H7.1 and NFKB1. Subsequently, we performed RIP assays based on Ago2, which can enrich for targets bound by miRNAs upon immunoprecipitation. The AGO2-RIP assay can determine whether the lncRNA RP11-86H7.1 or NFKB1 is found in the same RNA-induced silencing complex (RISC) as miR-9-5p. We overexpressed lncRNA RP11-86H7.1 in 16HBE cells then pulled down Ago2 using an anti-Ago2 antibody. Overexpression of lncRNA RP11-86H7.1 caused a significant decrease in the enrichment of NFKB1 pulled down by Ago2 (Fig. 6C), indicating that there were less miRNA-bound NFKB1 present. The above results suggested that lncRNA RP11-86H7.1 function as a ceRNA by sponging miR-9-5p and regulated NFKB1 expression indirectly.

### LncRNA RP11-86H7.1 acts as a ceRNA by sponging miR-9-5p to upregulate their common target, NFKB1 and promote inflammatory response

**Figure 7:**
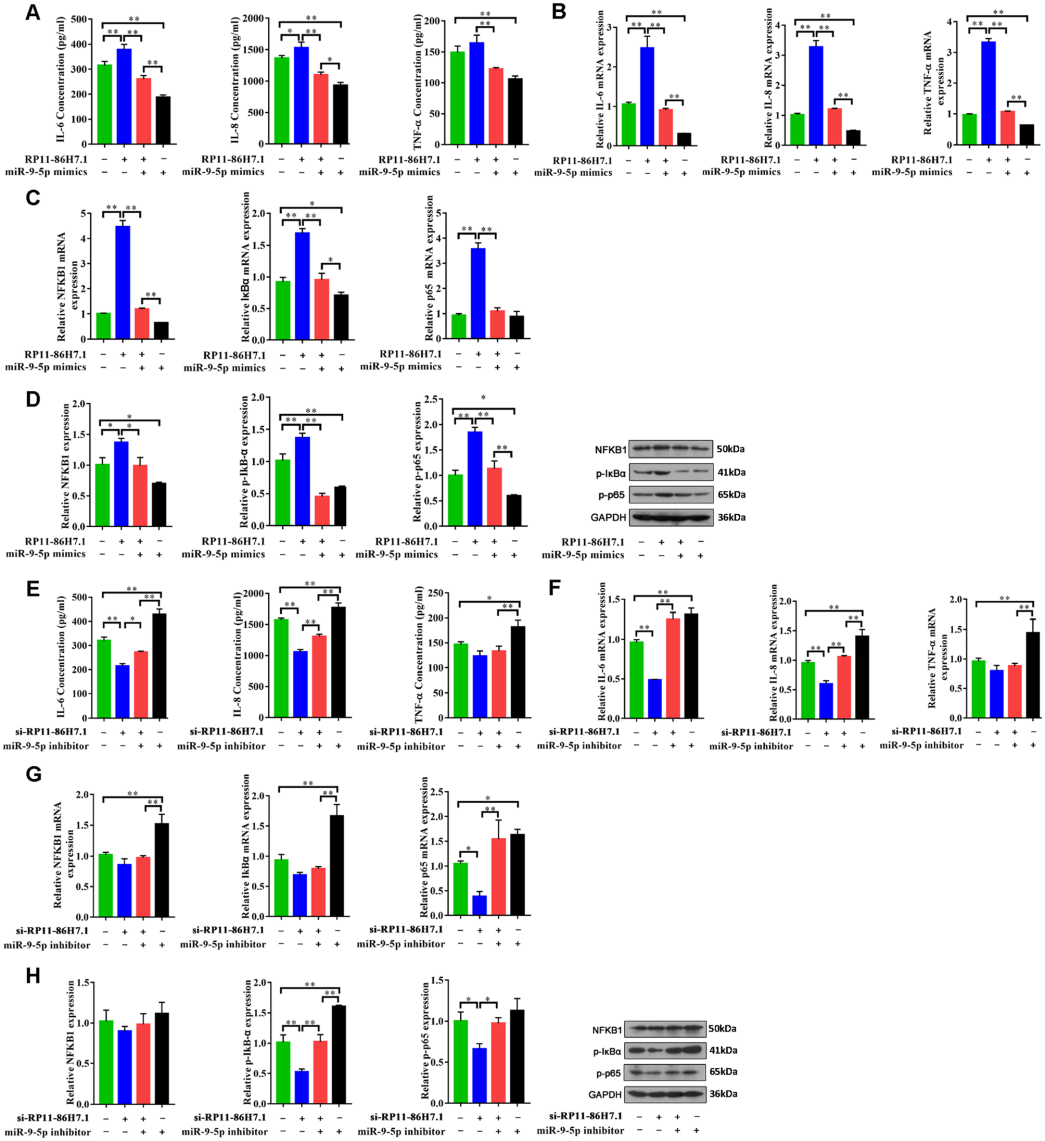
LncRNA RP11-86H7.1 acts as a ceRNA by sponging miR-9-5p to upregulate their common target, NFKB1 and promote inflammatory response by the gain and loss of function assay. (**A**) ELISA analysis of IL-6, IL-8, and TNF-α levels in 16HBE cells co-transfected with lncRNA RP11-86H7.1 or miR-9-5p mimic. (**B, C**) Q-PCR analysis of the mRNA expression levels of IL-6, IL-8, TNF-α, lncRNA RP11-86H7.1, NFKB1, IκBα, and p65 in 16HBE cells co-transfected with lncRNA RP11-86H7.1 or miR-9-5p mimic. (**D**) Western blotting analysis of the protein levels of NFKB1, p-IκBα and p-p65 in 16HBE cells co-transfected with lncRNA RP11-86H7.1 or miR-9-5p mimic. GAPDH was used as an internal control. (**E**) ELISA analysis of IL-6, IL-8, and TNF-α levels 16HBE cells co-transfected with lncRNA RP11-86H7.1 knockdown or miR-9-5p inhibitor. (**F, G**) Q-PCR analysis of the mRNA expression levels of IL-6, IL-8, TNF-α, lncRNA RP11-86H7.1, NFKB1, IκBα, and p65 in 16HBE cells co-transfected with lncRNA RP11-86H7.1 knockdown or miR-9-5p inhibitor. (**H**) Western blot analysis of the protein levels of NFKB1, p-IκBα and p-p65 in 16HBE cells co-transfected with lncRNA RP11-86H7.1 knockdown or miR-9-5p inhibitor. GAPDH was used as an internal control. The data are shown as the means ± standard deviations (n = 3). The statistical significance of the data was assessed by Student’s t test. *P < 0.05, **P < 0.01.

In order to test the biological functions of lncRNA RP11-86H7.1 as ceRNAs on inflammatory response, we stably overexpressed or knocked down lncRNA RP11-86H7.1 in 16HBE cells to perform gain and loss of function studies. lncRNA RP11-86H7.1 overexpression led to upregulation of NFKB1 and enhanced the inflammatory response. When we explored its effect using ELISA, Q-PCR, and Western blotting assay, the expression levels of NFKB1, IL-6, IL-8, TNF-α, p65, IκBα, p-IκBα and p-p65 were up-regulated. Not surprisingly, miR-9-5p mimics could abolish these effects caused by lncRNA RP11-86H7.1(Fig. 7A–D). In contrast, knockdown of endogenous lncRNA RP11-86H7.1 expression dramatically reduced the inflammatory response of 16HBE cells. As expected, inhibition of miR-9-5p could again decrease these effects (Fig. 7E–H).

## Discussion

Increasing studies have shown that long-term exposure to ambient air pollution increases the morbidity and mortality caused by COPD, shortens the life expectancy^26^ and is a leading contributor to the global disease burden. In particular, ambient PM2.5 was the fifth-ranked mortality risk factor in 2015^27^. Numerous epidemiological studies have revealed that PM2.5 from diesel-powered vehicles and from the tires and brakes of motor vehicles (TRAPM2.5) can increase the incidence of COPD, the risk of acute exacerbation, and the mortality rate and decrease lung function^9–14^. A recent study conducted by our research group provides direct evidence showing that chronic exposure to motor vehicle exhaust induces pulmonary changes in rats consistent with those observed in COPD lungs, including pulmonary inflammation, airway remodeling and emphysema^3^.

It is well known that airway inflammation caused by harmful particles or gases is an important factor in the pathogenesis of COPD^6^. Some studies have shown that the NF-κB pathway is closely related to COPD. NF-κB, IL-6, IL-8 and TNF-α, all of which have the characteristics of high potency and synergistic and mutual induction, resulting in the amplification of the inflammatory cytokine cascade, which can, in turn, lead to the production of oxygen free radicals, form a vicious circle, aggravate lung tissue, and induce persistence of the chronic inflammatory state of COPD. The genes encoding pro-inflammatory mediators, including IL-1, IL-6, IL-8, MCP-1, and TNF-α, all of which are regulated by NF-κB, are involved in the inflammatory process of the airways in COPD^22–24^. This finding suggests that NF-κB activation plays an important role in the chronic inflammatory response observed in COPD. In our study, to examine the effect of TRAPM2.5 on airway inflammation and to elucidate the underlying mechanisms, we first showed that TRAPM2.5 we collected from traffic jams induced the inflammatory response related to IL-6, IL-8, and TNF-α in HBECs and 16HBE cells. NF-κB plays a key regulatory role in the PM-induced inflammatory response and is closely related to IL-6, IL-8 and TNF-α^22–24^. In mammals, five members of the NF-κB protein family have been identified, designated as RelA (p65), RelB, c-Rel, NF-κB1 (p105/p50) and NF-κB2 (p100/p52), and the five members form homo- or heterodimers^28^. Under the resting state, NF-κB dimers are bound to inhibitory IκB-α proteins, thereby isolating the NF-κB complex in the cytoplasm. Various inflammatory stimuli such as LPS or TNFα can activate the IκB kinase (IKK) complex, consisting of IKKα, IKKβ, and IKKγ, which in turn phosphorylates IκB-α, thereby releasing the bound NF-κB dimers to transfer to the cell nucleus^29^. We subsequently observed the protein levels of IKKβ, p-IκB-α and p-p65 were significantly increased after treatment with TRAPM2.5 for 48 h. These results indicated that TRAPM2.5 induced the expression of inflammatory factors in HBECs through activation of the NF-κB signaling pathway.

Accumulating lines of evidence obtained in recent years suggest that lncRNAs are active and important biological molecules rather than “transcriptional noise”^30^. One hundred twenty and 43 lncRNAs are overexpressed and underexpressed in COPD patients, respectively, compared with normal lung tissue^31^. The mechanism underlying the impact of these lncRNAs on airway inflammation and tissue injury in COPD is being actively investigated. We performed a gene microarray and bioinformatic analysis to identify the lncRNA RP11-86H7.1 and and GRM7-AS1, which were associated with the NF-κB signaling pathway, and verified its significant upregulation in HBECs after TRAPM2.5 stimulation. These findings suggest that these two lncRNAs might play a critical role in the biological process of airway inflammation. Successful construction and screening of siRNA interference fragments can provide an experimental basis for subsequent research. We revealed a significant interference effect on the interference fragment 3 of the lncRNA RP11-86H7.1(siRNA RP11-86H7.1-3). However, a significant induction of GRM7-AS1 with siGRM7-AS1-1 was shown in Fig. 3D, possibly due to the fact that the primers are not ideal and may cause errors in quantitative PCR. Primers should be designed upstream of the ATG or downstream of the stop codon to avoid sequences used for RNAi being also counted as mRNA expression. Therefore, the lncRNA RP11-86H7.1 was selected as the target lncRNA in the subsequent studies.

To the best of our knowledge, this study constitutes the first systematic evaluation of the role of lncRNAs in the inflammatory response induced by TRAPM2.5 in airway epithelial cells. In contrast, our results showed that knockdown of the lncRNA RP11-86H7.1 significantly decreased the expression of the inflammatory factors IL-6 and IL-8 and the protein expression of p65 and p-p65 induced by TRAPM2.5 in HBECs. Moreover, knockdown of the lncRNA RP11-86H7.1 inhibited the translocation of p65 into the nucleus, which demonstrated that the NF-κB signaling pathway was also suppressed. Therefore, our results suggest that the lncRNA RP11-86H7.1 plays a necessary role in the inflammatory response induced by TRAPM2.5 in vitro. We also observed knockdown of RP11-86H7.1 decreased TRAPM2.5-induced IL-6 and IL-8 expression but not TNF-α whereas miR-9-5p overexpression inhibited expression of all three cytokines as reported in a study^32^. This shows that during the increase of TNF-α expression induced by TRAPM2.5, lncRNA is not a key molecule or a direct target to regulate TNF-α, and the process may involve other molecules or mechanisms.

To better study the function of the lncRNA RP11-86H7.1, we obtained the full-length sequence of lncRNA RP11-86H7.1 using EMSA technology. To determine the protein-encoding ability of the lncRNA RP11-86H7.1, we subsequently inserted the full-length sequence of the lncRNA RP11-86H7.1 into the coding potential analysis website (https://cpc.cbi.pku.edu.cn) and predicted that it does not encode a protein, which is characteristic of noncoding RNAs. It is well known that microRNAs can silence their target genes by binding to mRNA 3′-UTRs, whereas ceRNAs, such as lncRNAs, can competitively bind microRNAs through microRNA response elements (MREs) to affect the silencing effect of microRNAs on downstream target genes^33^. This novel regulatory mechanism exists between lncRNAs and miRNAs. However, according to the ceRNA hypothesis, whether lncRNAs act as effective ceRNAs depends mainly on their abundance and subcellular localization in the cytoplasm. Our subsequent experiment demonstrated that the lncRNA RP11-86H7.1 was distributed in the cytoplasm. Therefore, we speculated that the lncRNA RP11-86H7.1 might act as a ceRNA by sponging miRNA and thus indirectly regulates mRNA expression. To further determine the role of the lncRNA RP11-86H7.1, we constructed a map of the lncRNA-miRNA-mRNA co-expression network using bioinformatics databases and found that four miRNAs (miR-17-5p, miR-221-3p, miR-31-5p, and miR-9-5p) were associated with the NF-κB signaling pathway and might interact with the lncRNA RP11-86H7.1. Among these miRNAs, miR-9-5p could bind to both the lncRNA RP11-86H7.1 and NFKB1. We then confirmed the regulatory relationship among the lncRNA RP11-86H7.1, miR-9-5p and NFKB1 based on the following findings: (1) knockdown of the lncRNA RP11-86H7.1 meaningfully boosted miR-9-5p expression and decreased NFKB1 expression; (2) through a dual-luciferase activity assay, the direct binding ability of the predicted miR-9-5p binding site on the lncRNA RP11-86H7.1 and NFKB1 was validated; (3) RIP assays revealed that the lncRNA RP11-86H7.1 is located in the same RISC as miR-9-5p and NFKB1; and (4) function rescue experiments demonstrated that miR-9-5p mimic can reverse the pro-inflammatory effect induced by overexpression of the lncRNA RP11-86H7.1 and decreased NFKB1 expression. Recently some studies have shown that NFKB1 is verified as a direct target gene of miR-9-5p and that miR-9-5p can negatively regulate NFKB1. MiR-9-5p over-expression can suppress the NF-κB signaling pathway through NFKB1 downregulation, thus alleviating inflammation and thrombosis in Deep Vein Thrombosis rats^32^. Another study indicates that down-regulation of LncRNA TUG1 decreased the levels of pro-inflammatory cytokines including TNF-α, IFN-γ, IL-6, and IL-17 by sponging miR-9-5p via targeting NF-KB1 in multiple sclerosis^34^.

There is compelling evidence suggesting that the lncRNA RP11-86H7.1 acts as a sponge for miRNA-9-5p and thereby hampers the inhibitory effect of this miRNA on NFKB1 in bronchial epithelial cells. Unfortunately, due to the species specificity of lncRNAs, we have been unable to validate our results in animal experiments.

**Figure 8:**
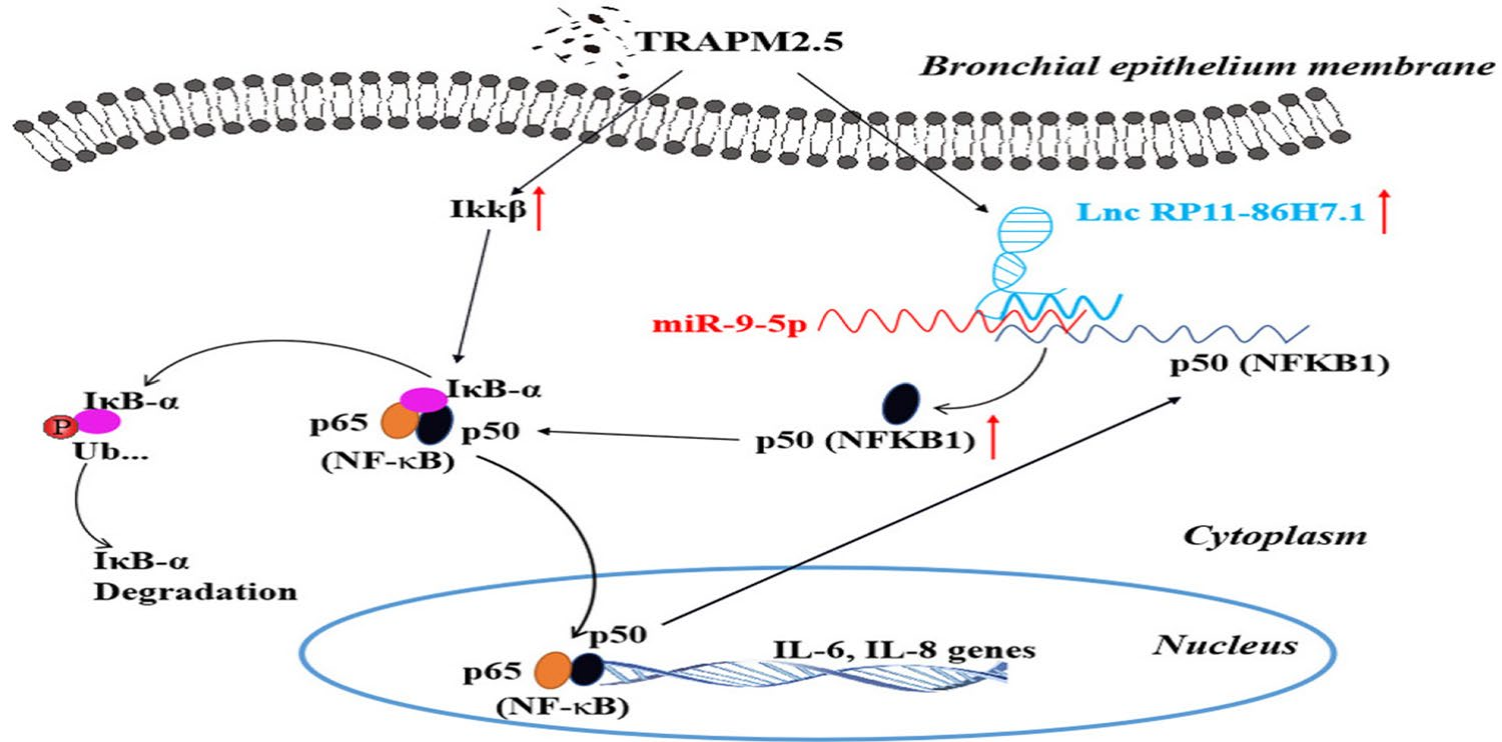
Schematic depicting the mechanism through which the lncRNA RP11-86H7.1 promotes TRAPM2.5-induced airway inflammation by acting as a ceRNA of miRNA-9-5p to regulate NFKB1 expression in bronchial epithelial cells, which is drawn using Microsoft PowerPoint 2019 software (https://www.microsoftstore.com.cn/office/office-home-student-2019/p/79g-05015).

In conclusion, our study provides evidence showing that the lncRNA RP11-86H7.1 promotes the inflammatory response of bronchial epithelial cells after TRAPM2.5 stimulation by acting as a ceRNA for miR-9-5p because the forced expression of the lncRNA RP11-86H7.1 reduced the expression of miR-9-5p, thereby released NFKB1 and sustained NF-κB activation. (Fig. 8). Thus, understanding the underlying regulatory mechanisms is of important potential clinical value and might provide further insights regarding new prevention and treatment strategies for airway inflammatory diseases caused by PM2.5 exposure, such as COPD.

## Materials and methods

### Antibodies and reagents

Antibodies against Iκκβ (ab32135) and p65 (ab7970) and Alexa Fluor 555 Donkey anti-Rb IgG (H + L) antibody (ab150074) were obtained from Abcam (Cambridge, MA, USA). The other antibodies used were as follows: anti-IKB-α (sc-1643) and anti-p-IKB-α (sc-8404) (Santa Cruz Biotechnology, CA, USA), anti-p-p65 (3033S; Cell Signaling Technology, Danvers, MA, USA), and anti-NFKB1 (NF-κB p50) (14220-1-AP; ProteinTech, Rosemont, IL, USA). Anti-GAPDH (H301; TransGen Biotech, Beijing, China) was used as normalized control. Enzyme-linked immunosorbent assay (ELISA) kits for IL-6 (CSB-E04638h), IL-8 (CSB-e04641h), and TNF-α (CSB-EQ023955HU) were obtained from Cusabio Biotech (Wuhan, China). M-MLV Reverse Transcriptase (M1701) and GoTaq qPCR Master Mix (A6001) were obtained from Promega (Madison, WI, USA).

### TRAPM2.5 collection and preparation

We used a high-flow sampler (TE-6070, Tisch, USA) equipped with a PM2.5 selective inlet head (1.13 m^3^/min) to collect TRAPM2.5 samples near Dongfeng West Road in Guangzhou, China, where traffic jams frequently occur, from 8:00 to 21:00 during July to September in 2015. Each filter membrane was collected after 13 h. TRAPM2.5 was collected on glass fiber membrane filters with a 1.6 μm pore size and a 406 cm^2^ sampling area, as previously described^18^. The amount of PM2.5 was defined as the increase in the weight of each filter. The filters were soaked in dimethyl sulfoxide (DMSO), and the solutions were then filtered through a 5-μm needle filter. The supernatant was collected, and the particles recovered from different filters were pooled to ensure a uniform batch of particles. The solutions of 12.5 mg/ml TRAPM2.5 were stored at − 20 °C until use. As the control, filters without particles were prepared under the same conditions using solutions containing 0.1% DMSO. The final concentration of DMSO in each well did not exceed 0.5%. The presence of DMSO had no effect on cell viability.

### Cell culture and treatment

Primary human bronchial/tracheal epithelial cells (HBECs) were purchased from American Type Culture Collection (ATCC, Manassas, VA, USA). The cells at passage 0 were seeded in 25 cm^2^ culture dishes in complete bronchial/tracheal epithelial cell growth medium (ATCC) and passaged once they reached 80% confluence. The 16HBE cell line was obtained from BeNa Culture Collection (Beijing, China) and cultured in Dulbecco’s modified Eagle’s medium (DMEM) supplemented with 10% fetal bovine serum (FBS). The HEK-293 T cell line was purchased from the Chinese Academy of Sciences Cell Bank (Shanghai, China) and cultured in DMEM supplemented with 10% FBS. All the cells were cultured in an incubator with 5% CO_2_ at 37 °C.

The cells were exposed to TRAPM2.5 at concentrations ranging from 0 to 200 μg/ml to determine their effect on cell viability, and the HBEC and 16HBE cells were exposed to 100 μg/ml TRAPM2.5 for 48 h for subsequent experiments. Extracts from clean filters containing 0.1% DMSO were used to prepare the controls.

### Microarray analysis

The three matched-paired sets of HBECs for the microarray analysis were obtained before and 48 h after TRAPM2.5 treatment, and total RNA was extracted from these cells. The Quick Amp Labeling kit (Agilent, USA) was used to amplify and transcribe the RNA into cRNA, and the labeled cRNA was then hybridized onto the 4** × **44 K Agilent Whole Human Genome Oligo Microarray (Agilent, USA) using the Gene Expression Hybridization Kit (Agilent, USA). The microarrays were washed with Gene Expression Wash Buffer (Agilent, USA) and scanned using an Axon GenePix 4000B microarray scanner (Molecular Devices, USA). The acquired microarray images were then extracted and analyzed using Agilent Feature Extraction Software v10.7. The original signal intensities were normalized using the quantile method with GeneSpring GX v11.5.1 (Agilent, USA), and the low-intensity lncRNAs and mRNAs were excluded. The box plot and scatter plot filters were used to identify differentially expressed lncRNAs and mRNAs. The thresholds used for the identification of up- or downregulated lncRNAs and mRNAs were fold-change > 2.0 and p value < 0.05.

### Enzyme-linked immunosorbent assay (ELISA)

The IL-6, IL-8 and TNF-α levels were measured using ELISA kits (Cusabio Biotech, China) according to the manufacturer’s protocols, respectively.

### Immunofluorescence

Immunofluorescence of p65 was detected according to the standard protocol^35^.

### RNA fluorescence in situ hybridization (FISH)

A FISH kit (Forevergen, China) was used according to the manufacturer’s recommended protocol, and the fluorescence was visualized using a confocal microscope (Zeiss, Oberkochen, Germany). 16HBE cells were seeded, fixed with 4% paraformaldehyde, treated with 0.5% Triton in phosphate buffer saline (PBS), and prehybridized. The cells were then hybridized overnight with the FAM probe at 5 μM. The labeled FISH probes were provided by BersinBio (Guangzhou, China). The probe sequences of lncRNA RP11-86H7.1 were provided in Supplementary Table S2.

### RNA extraction and quantitative real-time PCR

Total RNA was extracted using the Trizol reagent (Invitrogen, USA), and the RNA was then reverse-transcribed into cDNA in a final RT reaction volume of 10 µl using the M-MLV Reverse Transcriptase (Promega, USA). The cDNA (1.5 µl) was amplified by quantitative PCR with GoTaq qPCR Master Mix (Promega, USA) using an MXP3000 QPCR system (Stratagene, USA), and each reaction was performed in a volume of 20 µl. β-Actin and U6 snRNA were employed as endogenous controls for mRNA/lncRNA and miRNA, respectively. The relative mRNA amounts were calculated using the 2 − ^△△Ct^ method. The primer sequences used in this study are displayed in Supplementary Table S3.

### Protein isolation and Western blotting

The cells were lysed with RIPA buffer (Beyotime, China) on ice for 30 min, and supernatants were collected after three cycles of ultrasonography and centrifugation at 12,000×*g* and 4 °C for 20 min. The concentration of total protein was determined using the BCA Protein Assay Kit (Beyotime, China). Western blotting was proceeded as previously reported^36^. The protein expression levels were normalized against GAPDH expression.

### Transfection

Transient transfections were performed using the Lipofectamine 2000 Transfection Reagent (Invitrogen, USA) following the manufacturer’s recommended protocol.

### RNA interference

RP11-86H7.1, GRM7-AS1 and negative control (NC) siRNAs were designed and synthesized by Invitrogen Technologies. HBECs were grown in six-well plates to 50% confluence and then transfected with 50 nM siRNA using 7.5 μl of Lipofectamine RNAi MAX Reagent according to the manufacturer’s instructions. 24 h after transfection, the efficiency of siRNA knockdown was evaluated by Q-PCR.

### Rapid amplification of 5′ and 3′ cDNA ends (RACE)

Using total RNA extracted from HBECs, 5′ and 3′ RACE was performed to determine the transcription start points and the size of the lncRNA RP11-86H7.1 transcripts using a FirstChoice RLM-RACE kit (Ambion, USA) according to the manufacturer’s instructions. Due to the low copy number of the lncRNA RP11-86H7.1 in the cells, nested PCR was performed for each reaction. The primers used in this assay are displayed in Supplementary Table S4.

### Luciferase reporter assay

The wild-type and mutant RP11-86H7.1 fragment or NFKB1 3′-UTR containing the predicted binding sites of miR-9-5p were subcloned into a pmirGLO Dual-luciferase vector (Promega, USA). The luciferase reporter plasmids: pmirGLO-RP11-86H7.1-wt, pmirGLO-RP11-86H7.1-mut, pmirGLO- NFKB1-3′ UTR-wt, pmirGLO-NFKB1-3′ UTR-mut were co-transfected into 293 T cells with miR-9-5p mimics or the negative control (NC) by Lipofectamine 2000 transfection reagent (Invitrogen, USA) according to the manufacturer’s guidelines. Twenty-four hours after transfection, the 293 T cells were lysed using passive lysis buffer, and the relative luciferase activity was detected using the Dual-Luciferase Reporter Assay System (Promega, USA) and normalized to Renilla luciferase activity.

### AGO2-RNA immunoprecipitation (AGO2-RIP) assay

AGO2**-**RIP was performed using an Imprint RNA Immunoprecipitation (RIP) Kit (Sigma, USA) according to the manufacturer’s instructions. Briefly, the 16HBE cells were transfected with pcDNA3.1-lncRNA RP11-86H7.1, or pcDNA3.1. The samples were then incubated with RIP lysis buffer containing magnetic beads conjugated with Argonaute-2 (C34C6) Rabbit mAb (CST, USA). After the purified RNAs were extracted, real-time PCR was performed to examine the expression levels of lncRNA RP11-86H7.1 and NFKB1.

### Construction of lncRNA-miRNA-mRNA coexpression network

MiRNAda (https://www.microrna.org/microrna/home.do), TargetScan (https://www.targetscan.org), and miRcode (https://www.mircode.org/) were used to predict the interactions of the lncRNA RP11-86H7.1 with miRNAs. The miRNAs for the corresponding target genes were filtered out using the miRTarBase database (https://mirtarbase.mbc.nctu.edu.tw/php/index.php). The genes associated with the NF-κB signaling pathway were then identified using the KEGG database (https://www.kegg.jp). The mRNAs of the target genes associated with the NF-κB signaling pathway were subsequently filtered out. A map of the lncRNA-miRNA-mRNA co-expression network was drawn using Cytoscape v3.5.1 software (https://www.cytoscape.org/download.php).

### Statistical analyses

The statistical analyses were conducted using SPSS 16.0 software. All quantitative data are presented as the means ± standard deviations from at least three independent experiments. Differences between groups were analyzed using an unpaired Student’s t test. *p* values less than 0.05 were considered statistically significant.

## Supplementary information

Supplementary file1 (PDF 795 kb)

## Data Availability

The datasets generated during and/or analyzed during the current study are available from the corresponding author on reasonable request.
