# Supplementary material for: LncRNA RP11-86H7.1 promotes airway inflammation induced by TRAPM2.5 by acting as a ceRNA of miRNA-9-5p to regulate NFKB1 in HBECS
Source: Sci Rep. 2020 Jul 14;10:11587. doi: 10.1038/s41598-020-68327-1 (PMC7360621; doi:10.1038/s41598-020-68327-1)
Supplement: Supplementary file 1 — Supplementary file1 (PDF 795 kb) [file 41598_2020_68327_MOESM1_ESM.pdf]

Supplementary Information

LncRNA RP11-86H7.1 promotes airway inflammation induced by TRAPM2.5 by acting as a ceRNA of miRNA-9-5p to regulate NFKB1 in HBECS

Jun Zhao[1,2], Jinding Pu[1], Binwei Hao[1], Lingmei Huang[1,4], Jinglong Chen[1,2], Wei Hong[1,3], Yumin Zhou[1], Bing Li[3] and Pixin Ran[1,3] *

[1] State Key Laboratory of Respiratory Diseases, National Clinical Research Center for Respiratory Diseases, Guangzhou Institute of Respiratory Health, The First Affiliated Hospital of Guangzhou Medical University, Guangzhou, Guangdong, China

[2] Department of Geriatrics, national clinical key specialty, Guangzhou First People's Hospital, School of Medicine, South China University of Technology; Guangzhou First People's Hospital, Guangzhou Medical University, Guangzhou, Guangdong, China

[3]GMU-GIBH Joint School of Life Sciences, Guangzhou Medical University, Guangzhou, Guangdong, China

[4] The First People's Hospital of YueYang, YueYang, Hunan, China.

Figures

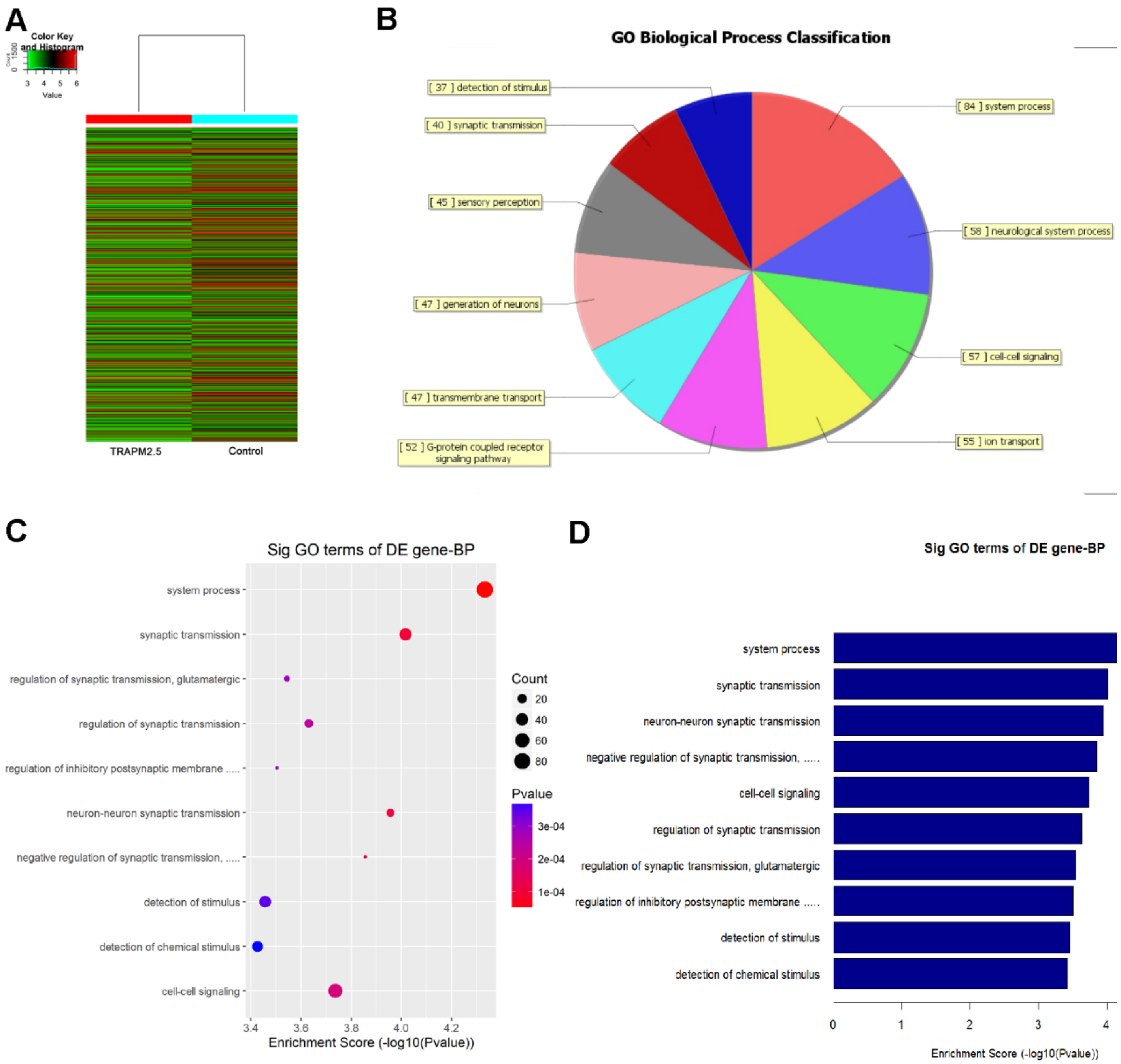

Supplementary Figure 1

Differential expression profiles of lncRNAs and mRNAs in HBECs after TRAPM2.5 treatment and bioinformatics analysis. (A) Heat map illustrating the lncRNA microarray data for HBECs before and 48 h after TRAPM2.5 treatment. The red color indicates high expression, and the green color indicates low expression. **(B)** Bioinformatics analysis of the biological processes associated with the mRNAs. **(C, D)** GO enrichment analysis of the molecular functions and cell compositions associated with the mRNAs.

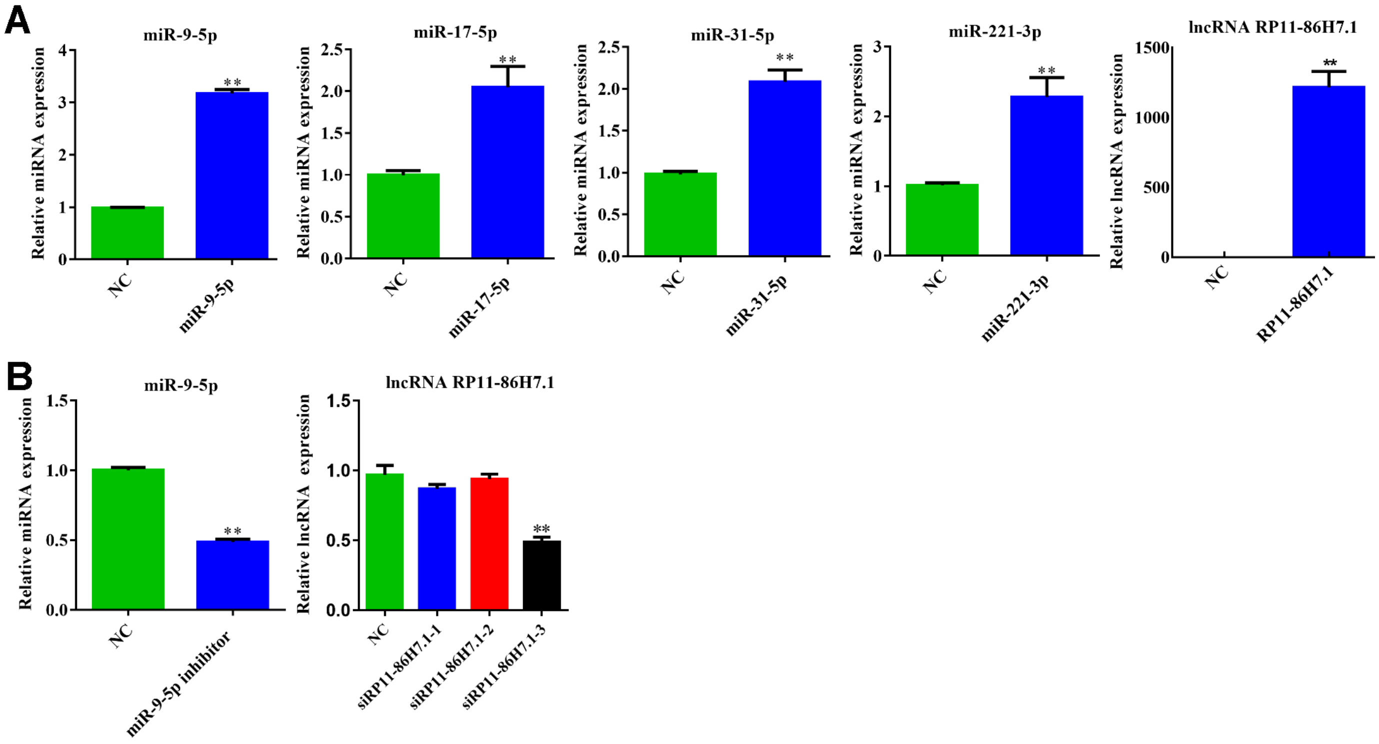

Supplementary Figure 2

Validate the effects of siRNA and mimics in miRNAs or lncRNA RP11-86H7.1 overexpression and knockdown experiments. (A) Q-PCR analysis of miR-9-5p, miR-17-5p, miR-31-5p, miR-221-3p, and lncRNA RP11-86H7.1 expressions levels after miRNAs (miR-9-5p, miR-17-5p, miR-31-5p, and miR-221-3p) and lncRNA RP11-86H7.1 mimic transfection. **(B)** Q-PCR analysis of miR-9-5p and lncRNA RP11-86H7.1 expressions levels after miR-9-5p inhibitor and lncRNA RP11-86H7.1 knockdown. The data are shown as the means ± standard deviations (n = 3). The statistical significance of the data was assessed by Student's t-test. **P < 0.01.

Supplementary Figure 3：**Original western blots images**

Figure 1B

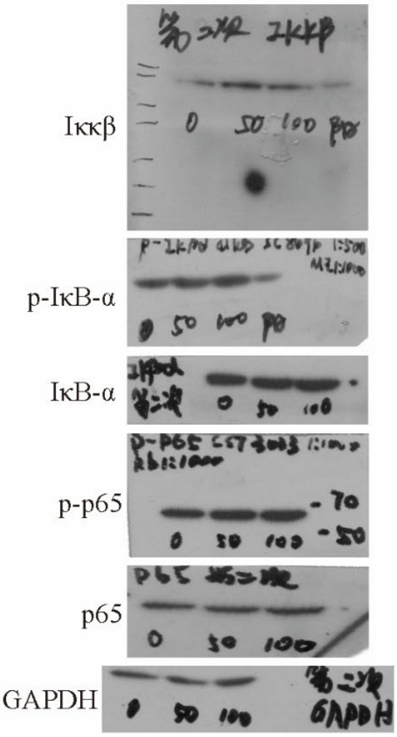

Figure 3D

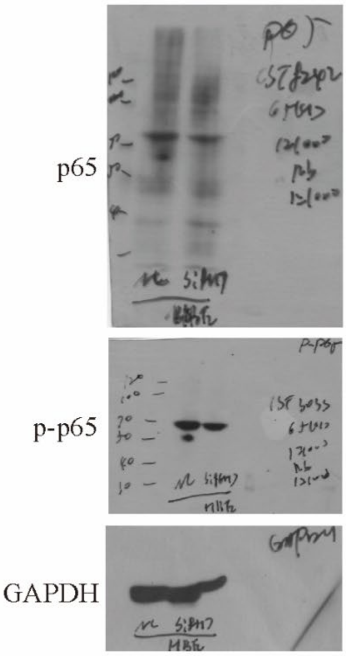

Figure 5D

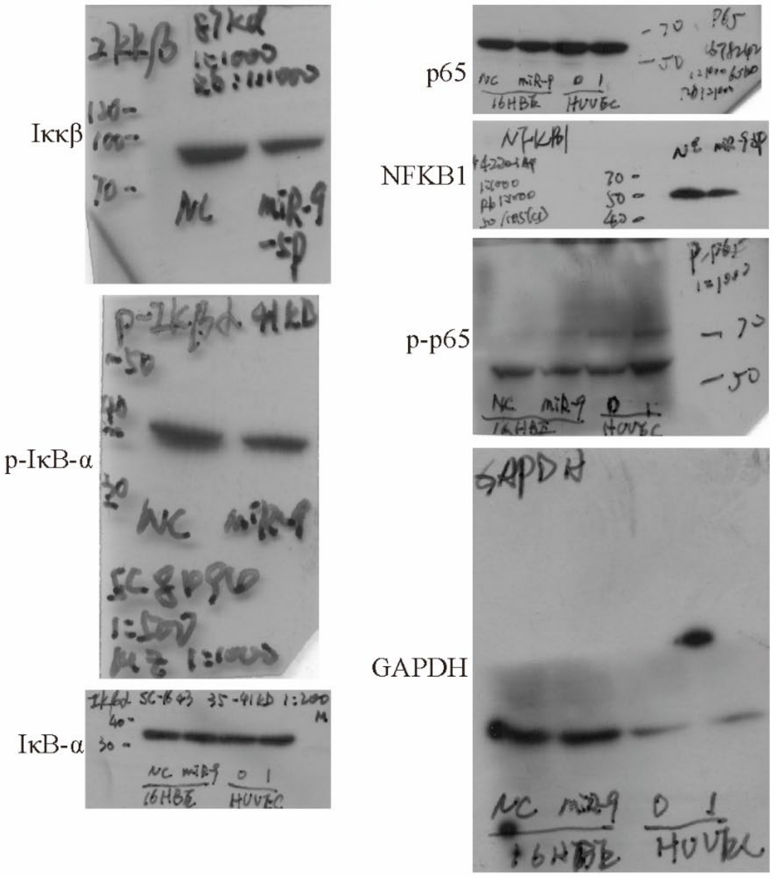

Figure 7D

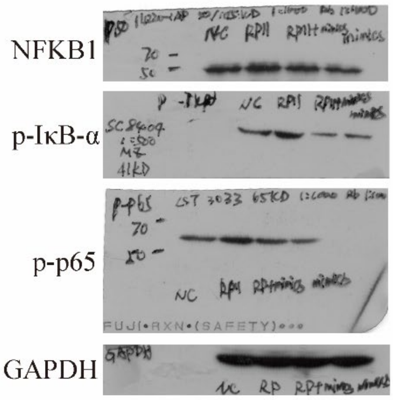

Figure 7H

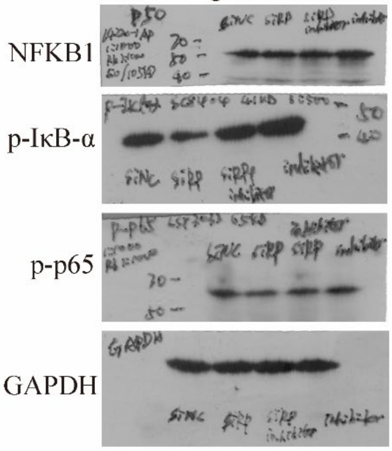

Tables

Table 1. Full-length gene sequence of the lncRNA rp11-86H7.1

Gene sequence of the lncRNA rp11-86H7.1
GCTGCGGTCCACTGGGTCCGCCTCTTCCCGGCATGGGTTTCTGCCCAGCT<br>CACCCGCGGTCAGCTGGGGTCCTGCTCTGGTGGGAGGAAGAGGCTCAG<br>ACGCTTCCCTGCCCTCTCGCCTCAACCACCTCGAGGCAGCGGCTCCCAG<br>GATGTGCACTTTGACGACTAAAGCTGAGCCGGCGCCGCCACGACCTTGG<br>GCGGGTGGTCGGCCTCTGCCCTGAGCAGGAAGTAGAAAGTCTCAGCAG<br>ACCCTTCCTGAGGGCCGAGCAACAGTGTAGTGGCGTATTCCACATAGCA<br>AACAGATGTACACTAGATTTTGATGGCATTTTTCCAGGGACCCTGGGAGC<br>ATCTGTGTTCTCGTGTTCTTCTTGCCTGTGAGGCCTCTTCTCTCTGAGGAC<br>AGATGGTAGGACAGAGAGAAGACAGCAAATGGAAAACAAAGGCTGCTA<br>TTTTTTTGTGGGGCAGGCAGCATTTCTTCCTGTGACAATATGGTGTAAGA<br>CAAAATTGTGGCCTGCCATGAAGTTGTGAGAGAAAAGCATCCCTCATTTC<br>TTCAGGAAAGGCCACTGCCGTTCCCTCCGGCTCCTCTTCTCAGGAGTTTG<br>TGCATTCATGAGGTGAATTCCTTGGAAGATGAGGGCCAAGTCTTATTCTT<br>CTCAGTCTCTTCTGCATCTAGCAGAAGAATAATGGTTGCACAGAAAATCT<br>TGGTTCAGTGGTTGAGTGAATGAATGGATGAACAGGCTCTTGCACACAA<br>AGAAGAGACATGATGTATTTGCATCCTTCCCTGCTTCAGCCAGGTCCCCA<br>GACAGTCCCGGTGTGCACACTTCCCTGCTTCTGTTCATGATGTTCTCTGA<br>ACCTGGGATCACCTTTCCCCTTCCTCTTACCTGCCCGATCCTACTTGTCCT<br>CCCAGATTCAGTTTTCTGAAGCTCAGAGGGACACCTTGTATTGCTGGATG<br>ATAAAAACAGGAGCAAAGTGATGAAGTGCTGACAAGGCAACAATAGAA<br>CATGAGAGATTCACTGCTGTGTAGGAAGAGATCTTCGGTGACCATGTAGC<br>CTGAAGCTCTCATTTTGTCATCGAGGGACACCAAAACTCAGAACAGTGC<br>TAAGCTCCTACATCCTGTTCTGGAAGGACAATCCTGAGCACACGATGAGC<br>CATATAAACTTCAGACTGACGCTGAGTGAAAGAATGCTGGAAAAGCATC<br>ACAGGCCAGGGCAGCAAAGTCTTCAAGGTCATCCGTGCTCTGATGATGT

CACGCTTCTTCCCTGTCTGGAAGACATTTCCCCAAGAGCATACCACCAAC ATCAGGGAAATGGAATCCAACTGGTCGCTGCAAAGTTTGCTGAGCACAC AGTGATAAAGATGGCAAGAAGGTCCTGAGAGAAACGCAATATTTTTGTG CAGAATGGGATGTTCTGCCTTTGTCCCATGCTTTGAAATTTACCACATGA AAAATAAAATTAAATACTGATCATCAAAAAAAAAAAAAAAAAA

Table 2. The probe sequences of lncRNA RP11-86H7.1 used in FISH

The probe sequences of lncRNA RP11-86H7.1
TCAGGAAGGGTCTGCTGAGACTTTCTACTTCCTGCTCAGGGCAGAGGCC GACCACCCGCCCAAGGTCGTGGCGGCGCCGGCTCAGCTTTAGTCGTCAA AGTGCACATCCTGGGAGCCGCTGCCTCGAGGTGGTTGAGGCGAGAGGG CAGGGAAGCGTCTGAGCCTCTTC

Table 3. Primer sequences

Name	Primer (5'→3')
IL-6 Forward	ACTCACCTCTTCAGAACGAATTG
IL-6 Reverse	CCATCTTTGGAAGGTTCAGGTTG
IL-8 Forward	GACCACACTGCGCCAACAC
IL-8 Reverse	CTTCTCCACAACCCTCTGCAC
TNF-α Forward	CTGCACTTTGGAGTGATCGG
TNF-α Reverse	GCTTGAGGGTTTGCTACAACAT
NF-κB p65 Forward	TGGCCCCTATGTGGAGATCA
NF-κB p65 Reverse	GTATCTGTGCTCCTCTCGCC
IκB-α Forward	ACACCTTGCCTGTGAGCAGG
IκB-α Reverse	AGCACCCAAGGACACCAAAA
NFKB1 Forward	GGTAACTCTGTTTTGCACCTAGCT
NFKB1 Reverse	AGGCTATTGCTCATCATGGCTA
Iκκβ Forward	CGATGGCACAATCAGGAAACAGGT
Iκκβ Reverse	ATTGGGGTGGGTCAGCCTTCTC

RP11-86H7.1 Forward	GGGAGCATCTGTGTTCTCGT
RP11-86H7.1 Reverse	ACTTCATGGCAGGCCACAAT
β-Actin Forward	CATGTACGTTGCTATCCAGGC
β-Actin Reverse	CTCCTTAATGTCACGCACGAT
hsa-miR-221 Forward	GCAGCTACATTGTCTGCTG
hsa-miR-221 Reverse	GTCGTATCCAGTGCAGGGTCCGAGGTATTCGC ACTGGATACGACGAAACC
hsa-miR-31-5p Forward	GAGGCAAGATGCTGGCA
hsa-miR-31-5p Reverse	GTCGTATCCAGTGCAGGGTCCGAGGTATTCGC ACTGGATACGACAGCTAT
hsa-miR-9-5p Forward	GGGTCTTTGGTTATCTAGCTG
hsa-miR-9-5p Reverse	GTCGTATCCAGTGCAGGGTCCGAGGTATTCGC ACTGGATACGACTCATAC
hsa-miR-17 Forward	GCACTGCAGTGAAGGCAC
hsa-miR-17 Reverse	GTCGTATCCAGTGCAGGGTCCGAGGTATTCGC ACTGGATACGACCTACAA
hsa-U6 Forward	CTCGCTTCGGCAGCACA
hsa-U6 Reverse	AACGCTTCACGAATTTGCGT
Universe Reverse	GTGCAGGGTCCGAGGT
SiGRM7-AS1-1 Forward	GAGTGACACTCCTCTTTAT
SiGRM7-AS1-1 Reverse	ATAAAGAGGAGTGTCACTC
SiGRM7-AS1-2 Forward	GACAATGTGTTCCAACAAT
SiGRM7-AS1-2 Reverse	ATTGTTGGAACACATTGTC
SiGRM7-AS1-3 Forward	CCGTTCCACATGCTTTCTT
SiGRM7-AS1-3 Reverse	AAGAAAGCATGTGGAACGG
SiRP11-86H7.1-1 Forward	GCAGGCAGCATTTCTTCCT
SiRP11-86H7.1-1 Reverse	AGGAAGAAATGCTGCCTGC
SiRP11-86H7.1-2 Forward	CCTGAGCAGGAAGTAGAAA
SiRP11-86H7.1-2 Reverse	TTTCTACTTCCTGCTCAGG

SiRP11-86H7.1-3 Forward	GGCTCTTGCACACAAAGAA
SiRP11-86H7.1-3 Reverse	TTCTTTGTGTGCAAGAGCC
Negative control Forward	TTCTCCGAACGTGTCACGTTT
Negative control Reverse	ACGTGACACGTTCGGAGAATT

Table 4. Primers sequences used in RACE

	Name	Primer (5'→3')
Linker primer	5' adaptor	GCTGTCAACGATACGCTACGTAACGGCAT GACAGTGCCCCCCCCCCCCCCC
	3' adaptor	GCTGTCAACGATACGCTACGTAACGGCAT GACAGTGTTTTTTTTTTTTTTTTT
	5.3' outer	GCTGTCAACGATACGCTACG TAAC
	5.3' inner	GCTACGTAACGGCATGACAGTG
3' RACE	RC386-F1	TGCTTCAGCCAGGTCCCCAGACA
	RC386-F2	CTGAACCTGGGATCACCTTTCCCCT
5' RACE	RC386-R3	CCTCGAGGTGGTTGAGGCGAGA
	RC386-R4	GCAGGGAAGCGTCTGAGCCTCTT
	RC386-RT1	GAAGAAATGAGGGATGCT
	RC386-RT2	ACAATTTTGTCTTACACCATA
